# Establishing an immunocompromised porcine model of human cancer for novel therapy development with pancreatic adenocarcinoma and irreversible electroporation

**DOI:** 10.1038/s41598-021-87228-5

**Published:** 2021-04-07

**Authors:** Alissa Hendricks-Wenger, Kenneth N. Aycock, Margaret A. Nagai-Singer, Sheryl Coutermarsh-Ott, Melvin F. Lorenzo, Jessica Gannon, Kyungjun Uh, Kayla Farrell, Natalie Beitel-White, Rebecca M. Brock, Alexander Simon, Holly A. Morrison, Joanne Tuohy, Sherrie Clark-Deener, Eli Vlaisavljevich, Rafael V. Davalos, Kiho Lee, Irving C. Allen

**Affiliations:** 1 Department of Animal and Poultry Sciences, College of Agriculture and Life Sciences, Virginia Tech, Blacksburg, VA 24061 USA; 2 Department of Biomedical Engineering and Mechanics, Virginia Polytechnic Institute and State University, Blacksburg, VA 24061 USA; 3 Department of Biomedical Sciences and Pathobiology, Virginia-Maryland College of Veterinary Medicine, Blacksburg, VA 24061 USA; 4 Department of Electrical and Computer Engineering, Virginia Polytechnic Institute and State University, Blacksburg, VA 24061 USA; 5 Department of Large Animal Clinical Sciences, Virginia-Maryland College of Veterinary Medicine, Blacksburg, VA 24061 USA; 6 Department of Mechanical Engineering, Virginia Polytechnic Institute and State University, Blacksburg, VA 24061 USA; 7 Department of Small Animal Clinical Sciences, Virginia-Maryland College of Veterinary Medicine, Blacksburg, VA 24061 USA; 8 Graduate Program in Translational Biology, Medicine and Health, Virginia Polytechnic Institute and State University, Roanoke, VA 24016 USA; 9 Institute for Critical Technology and Applied Sciences Center for Engineered Health, Virginia Tech, Kelly Hall, Blacksburg, VA 24061 USA; 10 Department of Basic Science Education, Virginia Tech Carilion School of Medicine, Roanoke, VA 24016 USA

**Keywords:** Cancer, Cancer models

## Abstract

New therapies to treat pancreatic cancer are direly needed. However, efficacious interventions lack a strong preclinical model that can recapitulate patients’ anatomy and physiology. Likewise, the availability of human primary malignant tissue for ex vivo studies is limited. These are significant limitations in the biomedical device field. We have developed *RAG2/IL2RG* deficient pigs using CRISPR/Cas9 as a large animal model with the novel application of cancer xenograft studies of human pancreatic adenocarcinoma. In this proof-of-concept study, these pigs were successfully generated using on-demand genetic modifications in embryos, circumventing the need for breeding and husbandry. Human Panc01 cells injected subcutaneously into the ears of *RAG2/IL2RG* deficient pigs demonstrated 100% engraftment with growth rates similar to those typically observed in mouse models. Histopathology revealed no immune cell infiltration and tumor morphology was highly consistent with the mouse models. The electrical properties and response to irreversible electroporation of the tumor tissue were found to be similar to excised human pancreatic cancer tumors. The ample tumor tissue produced enabled improved accuracy and modeling of the electrical properties of tumor tissue. Together, this suggests that this model will be useful and capable of bridging the gap of translating therapies from the bench to clinical application.

## Introduction

Despite relatively low rates of occurrence, pancreatic cancer accounts for a disproportionately high rate of cancer associated death. Surgery may cure patients with Stage I disease; however, more than 75% of patients will present with borderline or unresectable disease (Stages II–IV)^1,2^. Chemotherapy and radiation treatment protocols result in limited responses and are rarely curative^3–6^. Thus, new therapies are direly needed. Over the last decade, tumor ablation strategies, such as radiofrequency, microwave, and cryoablation have emerged as promising therapeutic options for pancreatic cancer^7–9^. Prior to human trials, the ablation modalities developed to date for pancreatic cancer were originally developed using models based on data generated from healthy tissues, rodent models, and occasionally spontaneous tumors in veterinary patients. Unfortunately, there are major limitations with each of these approaches that have hindered tumor ablation device development and limited data necessary for accurate treatment planning in human patients. Thus, the lack of clinically and physiologically relevant pre-clinical pancreatic cancer models continues to be a significant limitation.

Murine models are the dominant in vivo model in the biomedical engineering and cancer biology fields and are essential in acquiring fundamental data. Mice are easy to house and handle, relatively inexpensive, share many genetic similarities to humans, and there are many transgenic and knock-out lines available to study specific disease mechanisms, proteins, and pathways^10–15^. While these advantages make murine models appealing, the gap between mice and humans is wide enough to prevent many of the devices and therapies developed solely in mouse models from being safe and effective in human patients. There are important anatomical differences between humans and mice. Humans are approximately 2,500 times larger than mice, which accounts for differences in many biological aspects, including metabolic rates, heart rates, and life expectancy^15–19^. Looking at carcinogenesis, mice develop malignant tumors more readily than humans, due to significant differences in DNA repair and telomerase regulation, among others^20^. In an attempt to bridge this gap, more researchers are using patient-derived tumor xenograft (PDX) models for oncological studies. In these models, human tumors are engrafted into immunocompromised mice, allowing the local microenvironment of the human tumor to be recapitulated in the mouse^11,21–24^. While the PDX model is an impressive improvement to mouse models, the anatomical differences between mice and humans still presents a barrier to clinical relevance.

The availability of abundant, high quality tumor tissue is a major limitation in the development of medical devices that require ex vivo modeling and validation. This is especially true in pancreatic cancer research, where human specimens from patients are typically highly limited and restricted to small biopsies. Likewise, while post-mortem tissue is more available, the quality of the tissue is often not ideal. While mice are excellent bio-incubators for human tissue and cell lines, the size of tumor tissue is often limited due to the relatively small size of the animal. We have found this to also be a significant limitation in the development of irreversible electroporation (IRE) based therapeutic strategies. While healthy porcine tissues and mouse models have been critical for therapeutic development, we have found the lack of appropriate pancreatic cancer tissues to be a limitation. It has long been known that electrical conductivity deviates from its physiological value once tissues are removed from the body, but most tissues remain minimally changed within 1 h of removal^25^.

Members of our research team co-invented IRE as a non-thermal tissue ablation modality capable of treating solid tumors^26^. IRE involves delivering a series of low energy, unipolar electric pulses through electrodes inserted directly into the tumor. The induced electric field distribution produces structural defects in the target cell membrane that cause cell death. IRE has been successfully adapted for the treatment of tumors considered, until now, inoperable due to their proximity to critical structures such as blood vessels, nerves, and ducts^27–30^.

In an effort to circumvent the limitations related to animal models of pancreatic cancer and further advance the optimization of IRE utilizing human pancreatic cancer tissue, we developed an immunodeficient porcine model that has proven ideal for human cell transplantation and xenograft studies^31–35^. Utilizing CRISPR/Cas9, we have previously generated pigs with targeted deletions of *RAG2/IL2RG*, and established that these pigs are immunodeficient and thrive under our unique gnotobiotic housing conditions^35^. This prior study utilized these pigs to study the pathogenesis of a human virus^35^. Expanding upon these previous studies, here we deploy the *RAG2/IL2RG* deficient pigs as a novel tool to study pancreatic cancer. For the present study, we describe the original proof-of-concept studies, utilizing these pigs to propagate the human Panc01 pancreatic cancer cell line for use in ex vivo IRE modeling. This strategy generated an ample supply of high-quality pancreatic tumor tissue for electrical property modeling and IRE assessments. We believe that this model is a promising step towards studying human cancer treatments in a highly-relevant system with the potential for significant clinical impact.

## Results

### Generation of immunodeficient pigs carrying targeted deletions in *RAG2/IL2RG* using the CRISPR/Cas9 system

**Figure 1:**
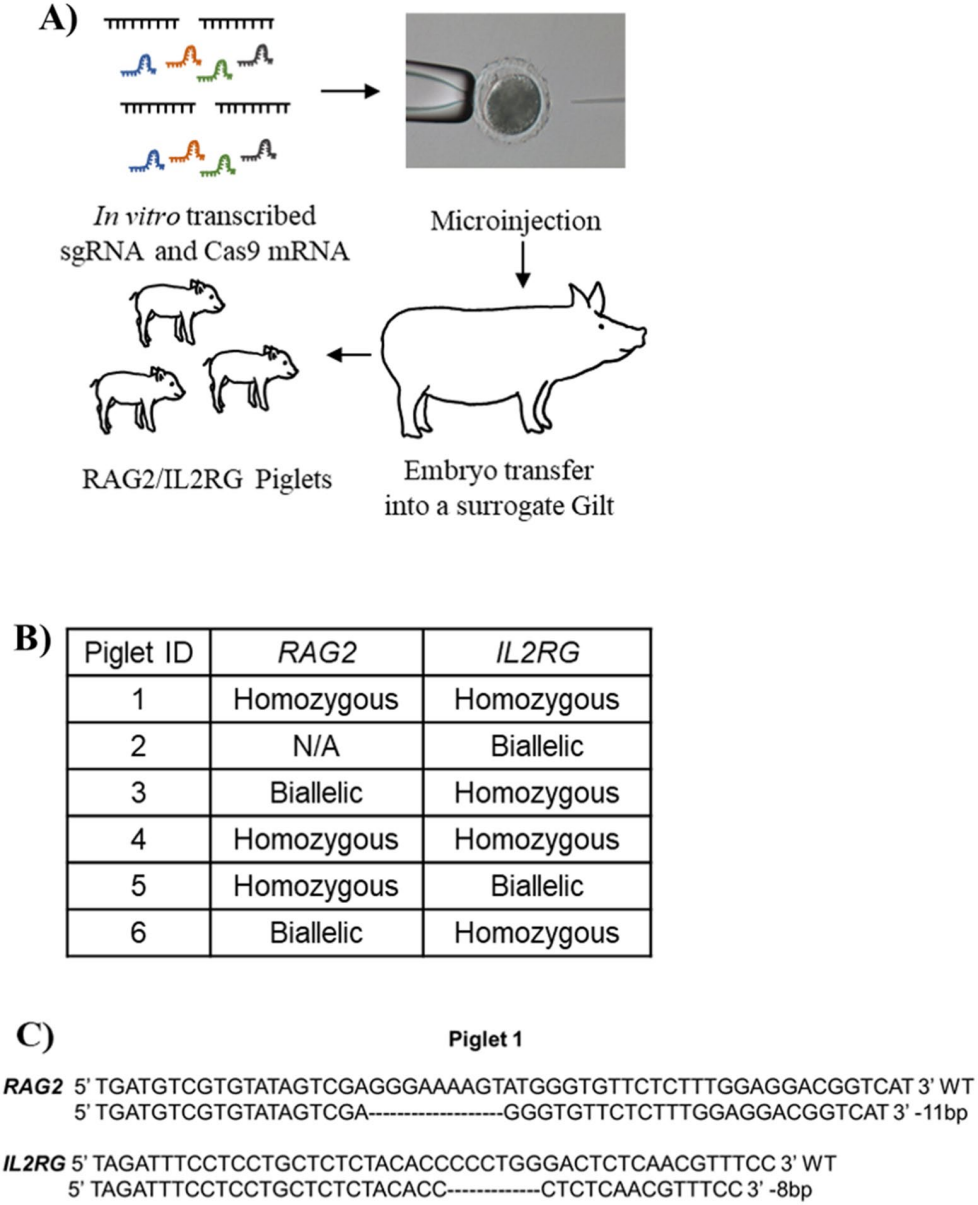
Generation of *RAG2*/*IL2RG* knockout pigs. (**A**) Schematic approach for *RAG2/IL2RG* knockout pig production. First, the sgRNA and Cas9 mRNA was synthesized via in vitro transcription. Second, an optimal concentration of sgRNA and Cas9 was injected into presumable zygotes. Third, the injected embryos were transferred into a surrogate gilt. At the end of gestation, piglets were born via hysterectomy and maintained in gnotobiotic isolators. (**B**) Summary of piglet genotypes in *IL2RG* and *RAG2* gene. Six piglets were born and analyzed. During PCR analysis, *RAG2* was not amplified from the Piglet #2 DNA sample, suggesting a large deletion. Biallelic genotype indicates two differently modified alleles. Homozygous indicates one type of modified alleles. (**C**) Genotype of Piglet 1 as a representation of small deletions or insertions introduced by the CRISPR/Cas9 system.

**Figure 2:**
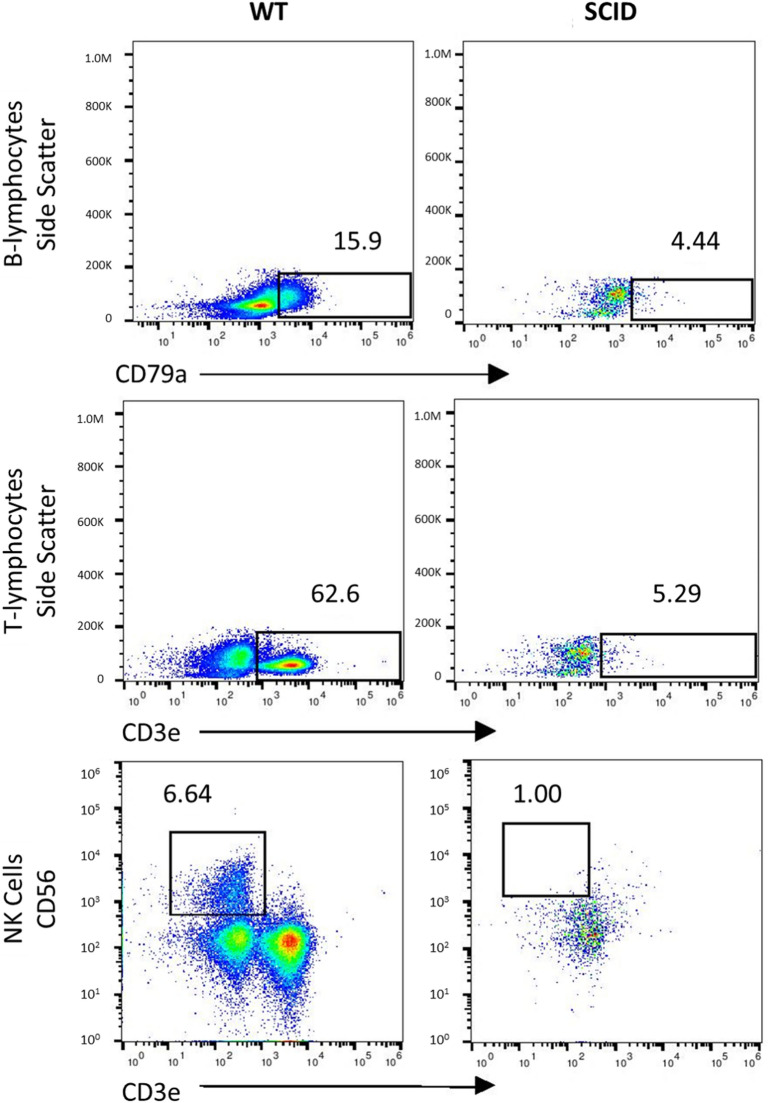
Confirmation of SCID-Like phenotype*.* FACS analysis to detect the presence of lymphocytes in *RAG2/IL2RG* knockout piglets. Compared to wild-type control, SCID piglet possessed lower level of B (CD79A), T (CD3E), and NK (CD3E-/CD56 +) cells.

Previous studies have utilized immunodeficient pigs in infectious disease studies and to evaluate engraftment of human iPS cells^35–37^. However, these pigs have yet to be widely utilized in cancer research models. CRISPR/Cas9 RNAs were introduced into the cytoplasm of in vitro fertilized pig oocytes (Fig. 1A). To disrupt pig immune system, the *RAG2* and *IL2RG* genes were targeted^35^. In vitro fertilized oocytes (n = 454) were injected with the targeted RNA for the CRISPR/Cas9 system, then cultured in vitro. On day 4 post-IVF, 155 embryos at between the 8-cell and morula stage were transferred to a surrogate gilt and subsequently six piglets were derived by hysterectomy. An important advantage of this strategy is the ability to generate the *RAG2/IL2RG* deficient pigs “on-demand”, without the need to maintain breeding colonies. Once born, the immunocompromised state of the pigs requires them to be maintained in gnotobiotic isolators under germ-free condition. Genotyping was conducted using genomic DNA collected from tail-snips. Similar to our previous report^35^, genotyping revealed that none of the six piglets have wild-type sequences in either of the *RAG2* or *IL2RG* genes (Fig. 1B detailed in Supplementary Fig. S1). All six piglets carried small insertion or deletion mutations in both *RAG2* and *IL2RG* genes (Fig. 1B). The genotype of piglet 1 is shown as an example (Fig. 1C). A *RAG2* fragment flanking the CRISPR/Cas9-induced DSBs site could not be amplified from one piglet by PCR, suggesting a larger deletion caused by CRISPR/Cas9 system. For tumor engraftment studies, 3 of the 6 pigs were randomly selected for subsequent Panc01 studies. Aligned with the RAG2/IL2RG knockout establishing study^35^, the piglets presented B-T-NK-SCID phenotype (Fig. 2).

### Panc01 tumors successfully engrafted in all pigs carrying the targeted RAG2/IL2RG deletion

**Figure 3:**
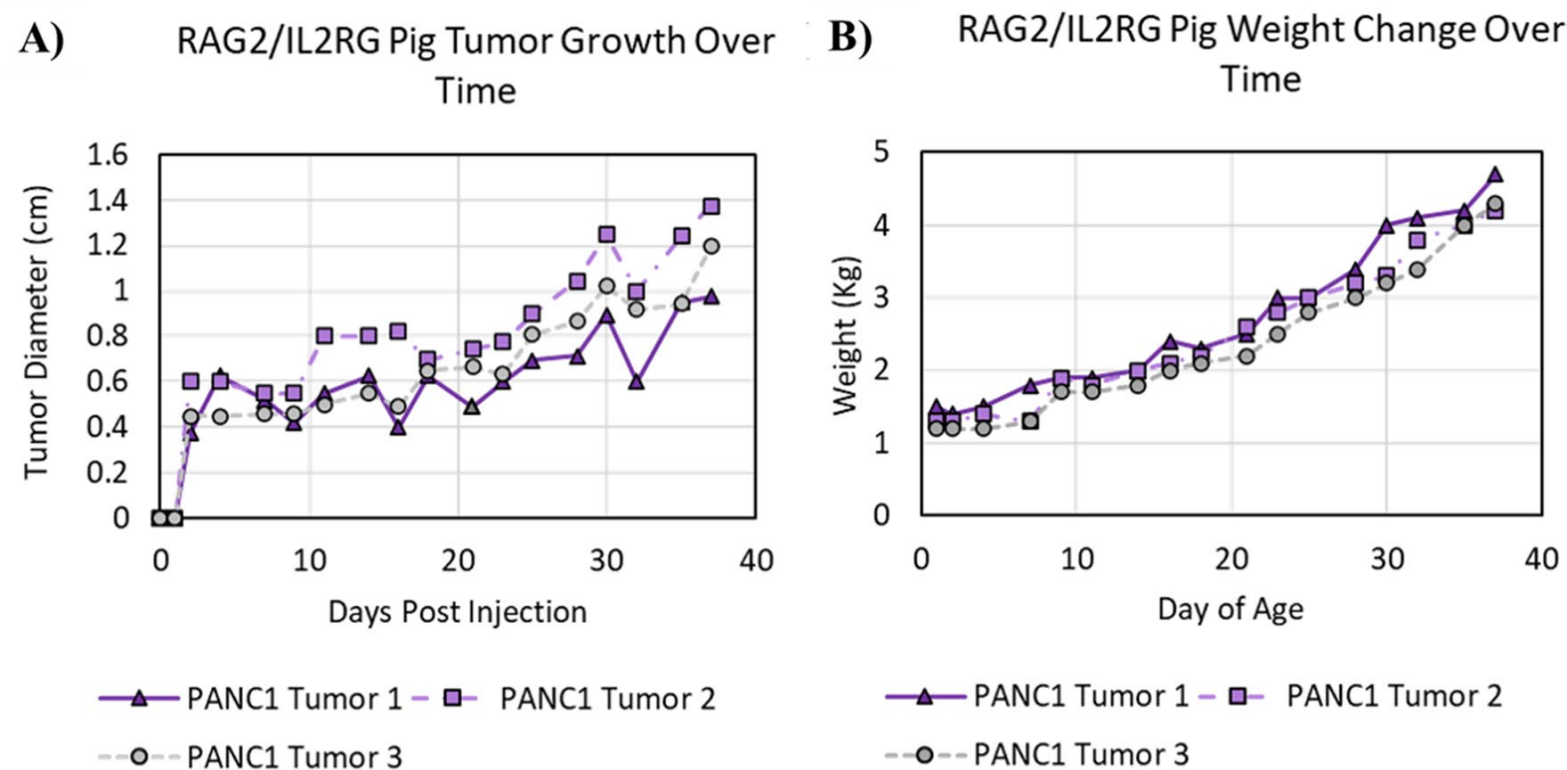
Successful engraftment of Panc01 cells. (**A**) Tumor growth was measured three times per week for each pig. All animals developed palpable tumors that continued to grow over the course of the study. (**B**) Pig weight was monitored throughout the duration of the study and utilized as a surrogate for animal morbidity.

**Figure 4:**
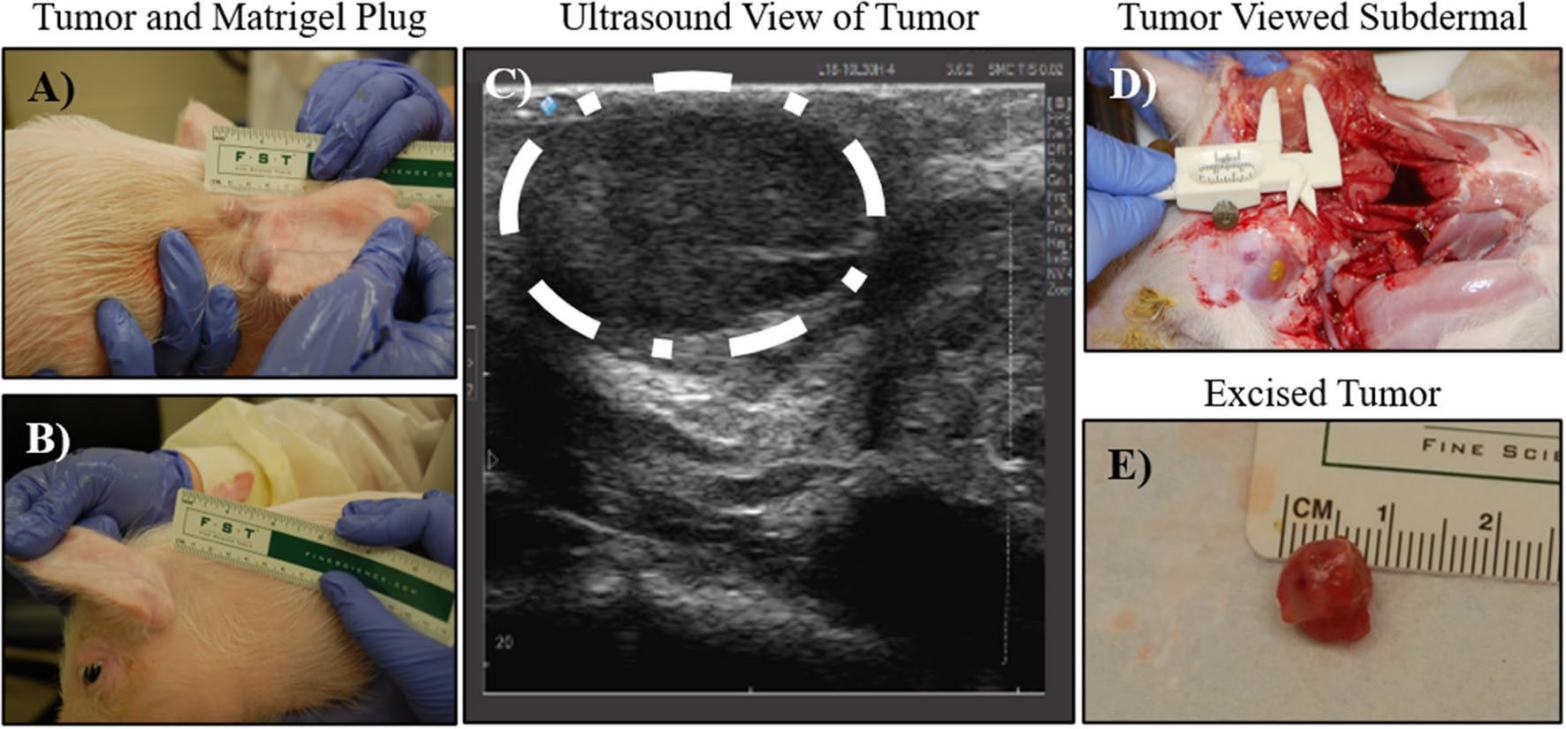
Subcutaneous tumors generated ample, high-quality pancreatic cancer tissue for subsequent ex vivo tumor ablation assessments. (**A**) Tumor growth was readily observable above the skin behind the ear where the Panc01 cells were injected. (**B**) No observable foci were located in regions where Matrigel alone (control) was injected. (**C**) Ultrasound image confirmed the volumetric growth of the tumors. The tumor is outlined here in white for clarity. (**D, E**) Necropsy of the tumors confirmed that (**D**) they were confined to the dermis and (**E**) approximately the same size as measured extra-dermally.

The purpose of the current study was to generate human tumors of sufficient size and quality in our unique immunocompromised pigs for ex vivo assessments of IRE. However, due to this being the first use of these pigs to propagate tumor tissue and the potential for incomplete knockout of the immune system due to inefficiencies in the CRISPR/Cas9 strategy, we first established engraftment proficiency and growth rates for the Panc01 xenografts. Within 24 h of the initial injection, tumor growth was observed for all Panc01 tumors. After an initial period of rapid growth, within 5 days of xenograft, the rate of increase stabilized and steadily increased to the target diameter of 1.0–1.6 cm by day 36 (Fig. 3A). No changes were observed in morbidity, mortality, or clinical parameters associated with tumor progression demonstrated here by the change in pig weight over time (Fig. 3B). All tumors were easily palpated and easily observed by day 36 (Fig. 4A). The Matrigel control injection was not detected (Fig. 4B). Additional assessments utilizing ultrasound imaging revealed clearly defined tumors, with clear delineations from surrounding tissues (Fig. 4C). Necropsy revealed the subcutaneous tumors remained within the dermal layer, and where easily palpated and observable from underneath the dermal layers (Fig. 4D). Tumor measurements collected extra-dermally, sub-dermally, and with ultrasound were comparable for each tumor (Fig. 4A, C–E). At gross necropsy, no evidence of metastasis was identified in draining or other lymph nodes, nor in organs with major vascular supply, such as the spleen, liver, or lung.

### Panc01 tumors are histopathologically similar to Pan02 and PDX tumors generated in mice

**Figure 5:**
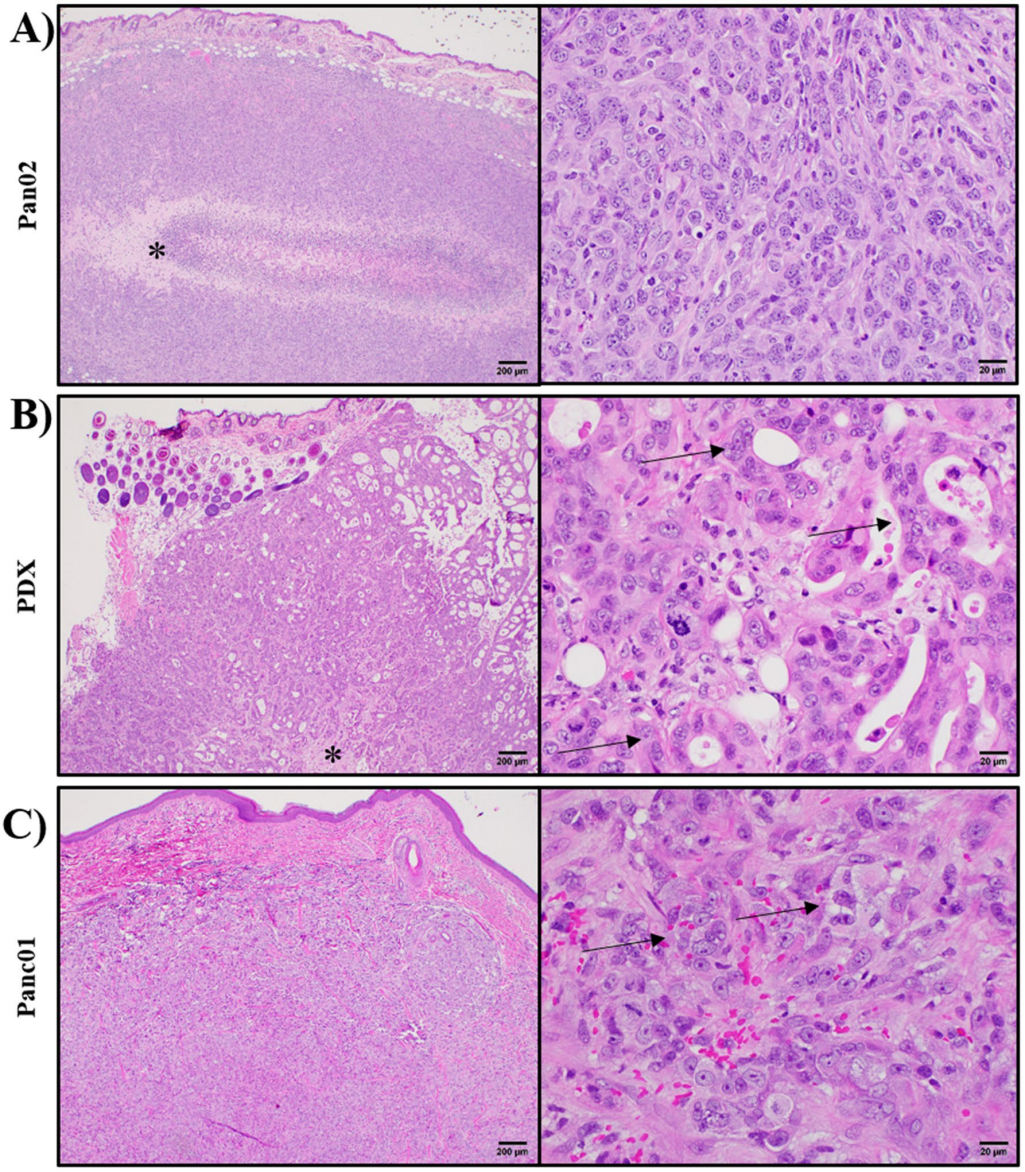
Histopathology comparison of pre-clinical pancreatic cancer animal models. mouse Pan02, human PDX, and human Panc01 were compared. (**A**) Subcutaneous tumors in the mouse Pan02 model are densely cellular often with central areas of necrosis (asterisk). Individual cells are spindle-shaped and arranged in vague, irregular streams. (**B**) Subcutaneous tumors in the PDX mice are also highly cellular and also exhibit foci of necrosis (asterisk). At higher magnification, these cells are more polygonal and are often arranged to form irregular tubular structures with lumens containing inflammatory cellsmurin and debris (arrows). (**C**) Subcutaneous tumors in the porcine Panc01 model are also densely cellular and variably exhibit foci of necrosis. Individual cells are more polygonal in shape and rarely attempt to recapitulate glandular structures (arrows).

**Figure 6:**
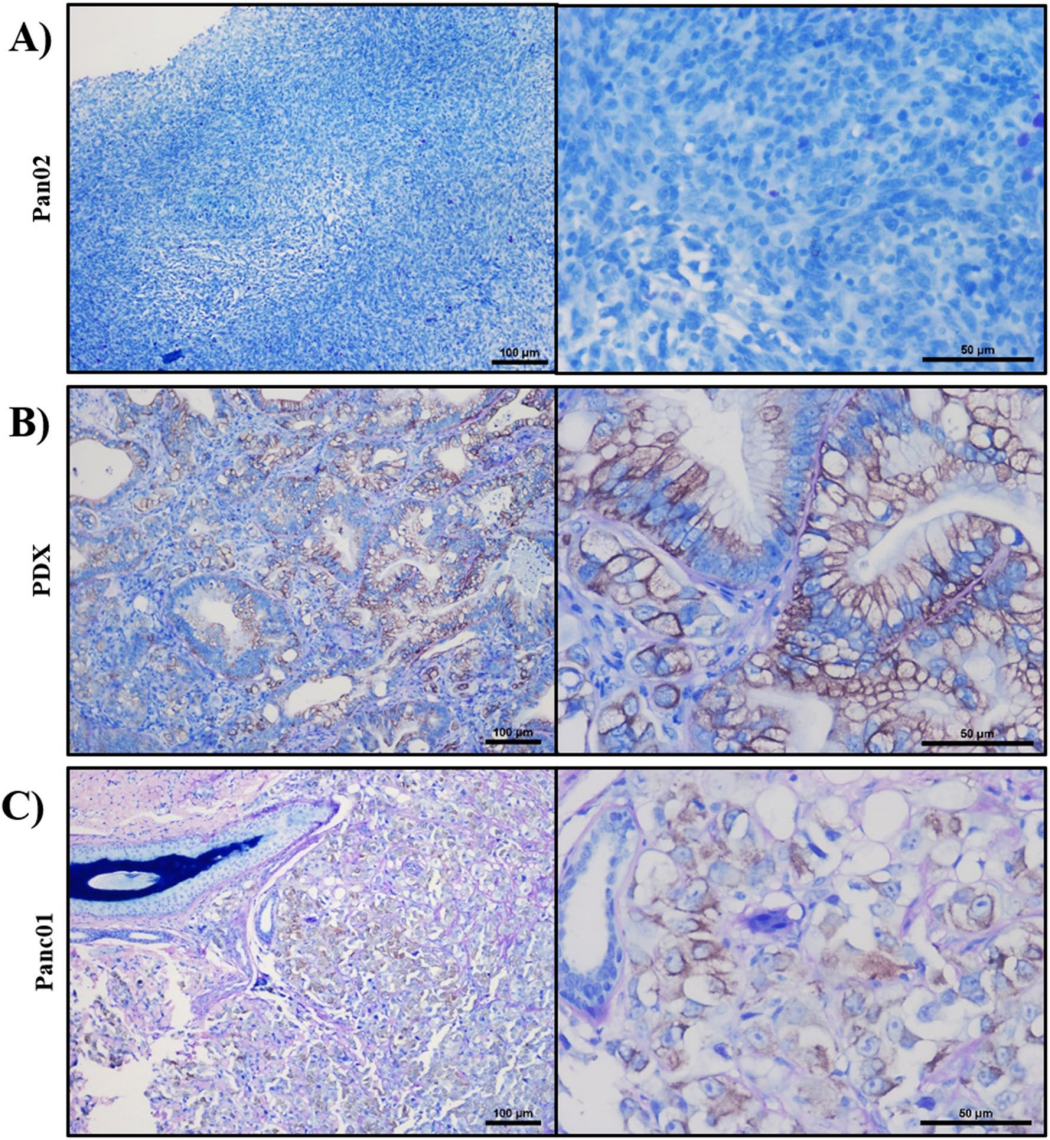
Immunohistochemical confirmation of ductal cells in pre-clinical pancreatic cancer animal models. (**A**) mouse Pan02, (**B**) human PDX, and (**C**) human Panc01 were compared for CK19 expression. Brown color indicates positive color. Nuclei are stained in dark blue, and remaining cell features are highlighted in light blue.

Due to the historic use of mice in IRE and other tumor ablation modality development^24,38–40^, we next compared the Panc01 tumors grown in this study from the *RAG2/IL2RG* pigs with Pan02 tumors generated in immunocompetent mice and PDX tissue derived tumor generated in immunocompromised mice. Previous studies have utilized human xenografts in immunocompromised mice or murine tumor injection into immunocompetent animals to generate tissue for ex vivo IRE studies. However, the small size of the mice is a restriction for tumor growth and limitation for any ultimate orthotopic studies. In general, cell line tumors from both animal models were highly similar. As expected, simply due to the immune system status of each animal, we did observe some minor differences in tumor histopathology. In the murine Pan02 and in the murine PDX subcutaneous pancreatic adenocarcinoma models, nodular tumors develop at the site of injection (Fig. 5A,B, respectively). The Pan02 tumors are densely cellular with minimal fibrovascular stroma and often variably-sized foci of necrosis develop centrally within the tumor. Tumor cells are spindle in shape and tend to be arranged in vague, indistinct streams. Similar to most malignant tumor cells of any origin, they often exhibit marked pleomorphism (differences in cell and nuclear size and shape across the population) with numerous mitotic figures scattered throughout. In the PDX model, cells derived from human tumors are injected into immunodeficient NOD-Scid gamma (NSG) mice. Microscopically, the tumors in these mice look different from those in the Pan02 model. For example, in the PDX model, tumor cells are more polygonal in shape (characteristic of epithelial cells) and are often arranged into disorganized glandular structures. In the *RAG2/IL2RG* pig xenografts of Panc01, microscopically the tumors are characterized by dense cellularity on minimal fibrovascular stroma, as seen in other models (Fig. 5C). Tumor cells, resemble an intermediate between the two murine models. Cells are more polygonal in shape and do rarely attempt to recapitulate glandular structures. These are often indistinct and do not often exhibit lumens. Localization of CK 19, an established ductal cell marker, was also analyzed in Pan02, PDX, and Pan01 tumors in order to establish the ductal characteristics of the tissues^41^. Murine Pan02 sections were not found to express CK19 (Fig. 6A). In the murine PDX and *RAG2/IL2RG* pig xenografts, CK19 expression was found throughout the tissues, localizing in glandular and glandular-like structures respectively (Fig. 6B–C).

### Panc01 tumors demonstrate similar electrical properties to primary patient derived pancreatic cancer tissue and healthy porcine tissues

**Figure 7:**
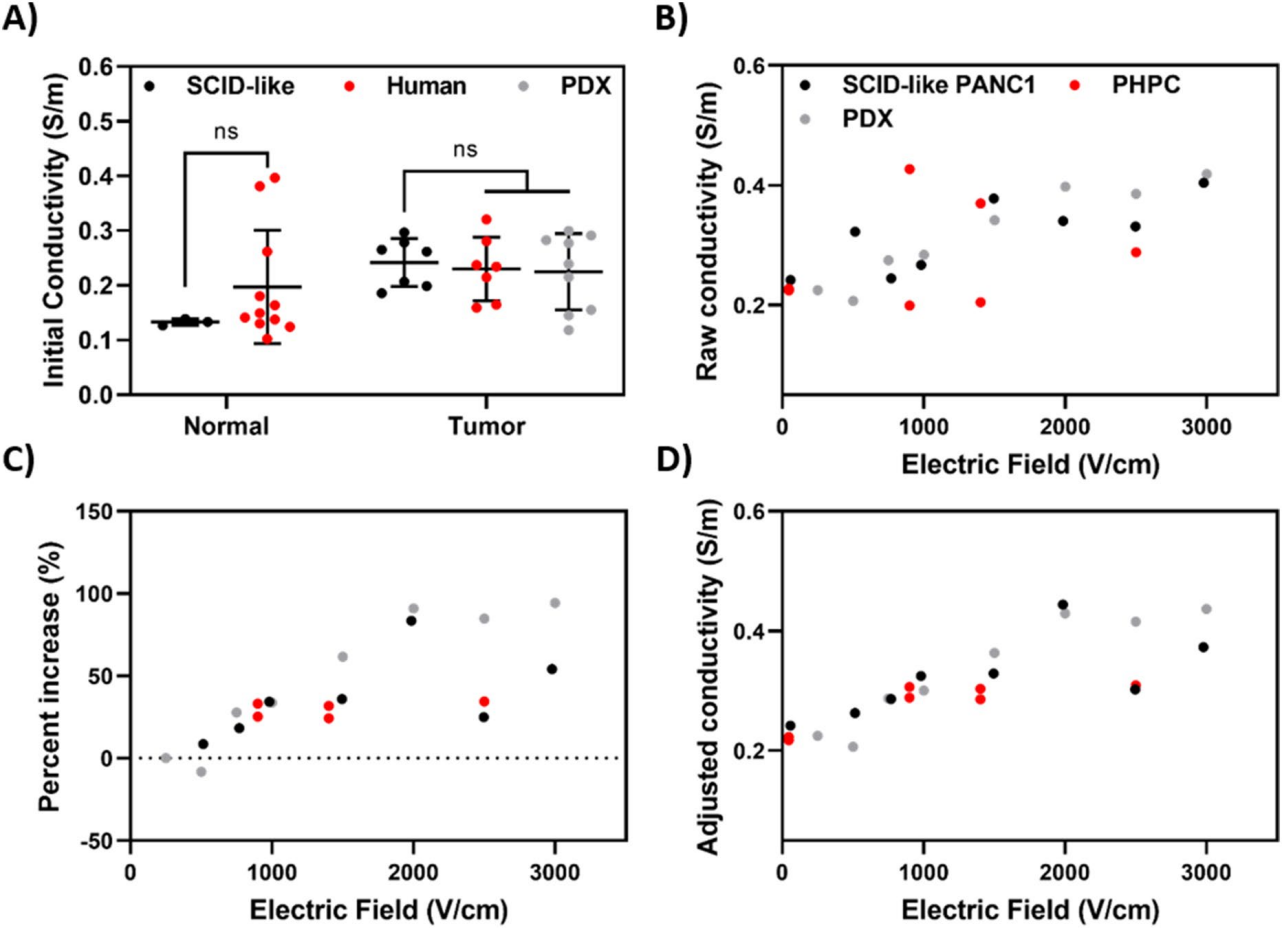
Primary human, SCID-like Panc01, and murine PDX tumors demonstrate similar conductivity increases following high voltage pulsed electric field. Ex vivo tissue samples directly from patients, *RAG2/IL2RG* animals, or NSG mice were sectioned and fit within a PDMS mold to impose a cylindrical shape factor. After sample preparation, individual samples were exposed to IRE pulses with varying electric field magnitudes. (**A**) Summary of initial conductivities calculated from normal and cancerous SCID-like (black), human (red), and murine PDX (gray) tissue. (**B**) The conductivity for each tissue type increased with the applied electric field. (**C**) Percent increase in tissue conductivity at varying fields was determined from the sample-specific pre-pulse values. (**D**) The adjusted conductivity is calculated from the percent difference values; here, the conductivities are normalized to the average initial tissue conductivity and adjusted based on the percent change.

There are several significant advantages of the Panc01 porcine xenograft model over other currently existing models and even primary human pancreatic cancer tissue. Specifically, the Panc01 tumors are readily available, high quality, and in sufficient volume for robust ex vivo studies. Thus, we next compared the electrical properties of the Panc01 tissue with primary human pancreatic cancer tissue (PHPC) and murine-propagated primary pancreatic ductal adenocarcinoma (PDAC) tissue as originally reported in Brock et al*.* (Fig. 7)^24^. The PHPC tissue (n = 7), excised Panc01 tumor tissue (n = 7), and murine PDX tissue (n = 9) exhibited initial conductivities of 0.231 ± 0.058 S/m, 0.242 ± 0.043 S/m, and 0.225 ± 0.070 S/m, respectively (Fig. 7A). No statistically significant differences were found between these values. With an applied electric field of 2.5 kV/cm, the mean intra-pulse conductivity increased to 0.288 S/m, 0.331 S/m, and 0.386 S/m for the PHPC, Panc01, and murine PDX tissue, respectively (Fig. 7B). The relationship between the treated tumors is more clearly seen as a percent increase from base line, with corresponding adjusted conductivity (Fig. 7C,D).

**Figure 8:**
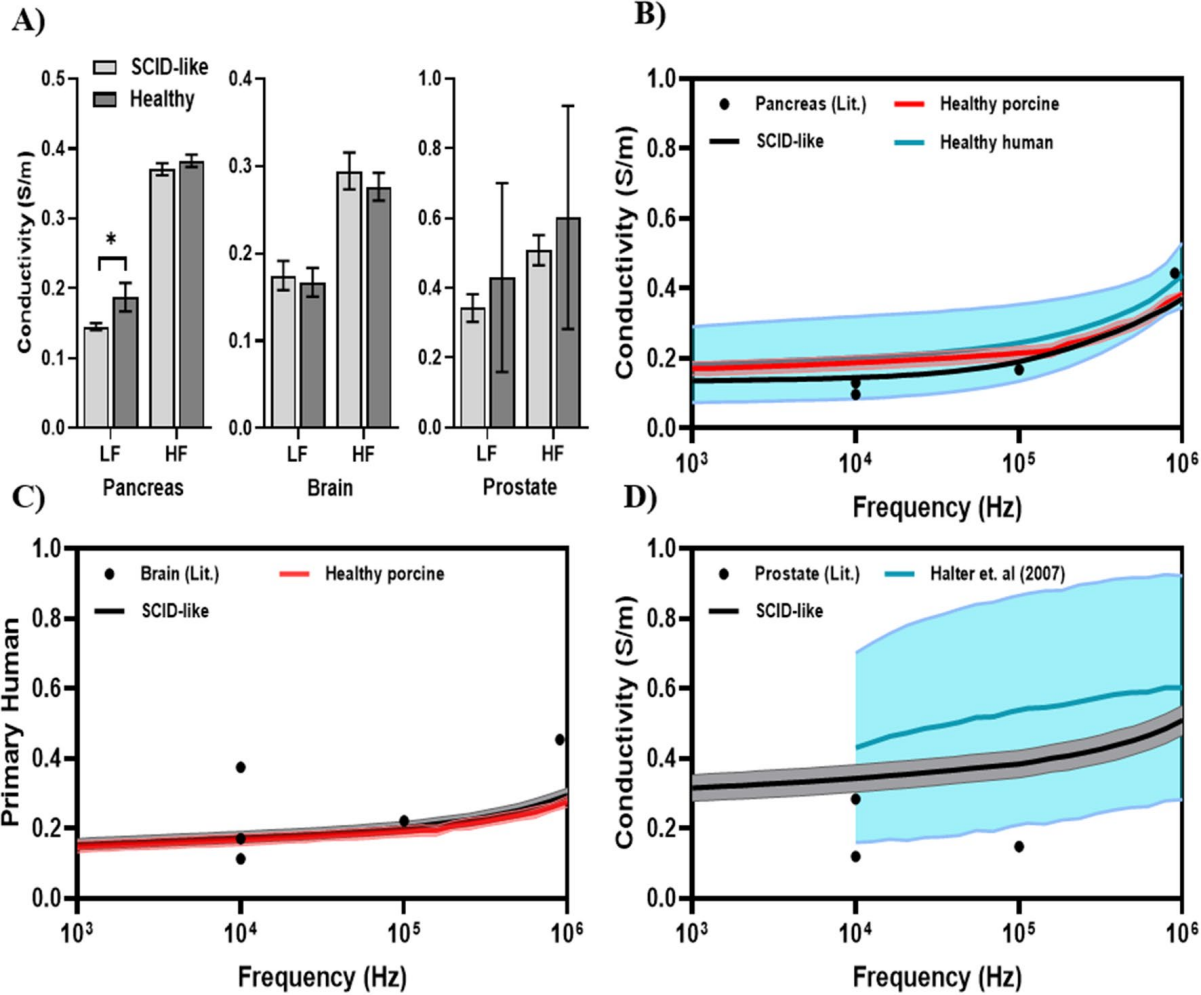
Impedance for healthy tissue excised from the *RAG2/IL2RG* pigs correlate adult swine and humans. A Gamry Reference 600 potentiostat (Gamry, Warminster, PA, US) was used to record tissue impedance within a frequency range of 10^3^–10^6^ Hz. The real part of the tissue impedance was used in the calculation of tissue conductivity for further comparison to values previously reported in literature. (**A**) Comparison of electrical conductivity from SCID-like pig tissue to healthy porcine (pancreas/brain) or human (prostate) tissue. The (**B**) pancreatic, (**C**) brain, and (**D**) prostate tissue demonstrate similar electrical behavior to their normal counterparts across a wide frequency range. Each data point (dot) represents reported values for normal tissue conductivity, while the black line represents data gathered in this study and the red line represents normal porcine tissue. (*p < 0.05).

Directly comparing the healthy porcine pancreas in *RAG2/IL2RG* pigs to human pancreas, the initial conductivity was 0.133 ± 0.0059 S/m and 0.197 ± 0.103 S/m (*p* = 0.0678), respectively (Fig. 7A). In addition to the healthy pig pancreas, we also compared results with healthy brain and prostate. The EIS results for healthy brain, pancreas, and prostate are reported as low frequency (10 kHz) and high frequency (1 MHz) measurements for direct comparison to literature values; the impedance spectrum for each tissue and the comparison to literature values are shown in Fig. 8. At low frequency, the conductivity values for healthy tissue analyzed from the *RAG2/IL2RG* model for brain (n = 8), pancreas (n = 3), and prostate (n = 7) tissue measured at 0.175 ± 0.017 S/m, 0.145 ± 0.005 S/m, and 0.343 ± 0.039 S/m. In this same frequency, the conductivity data measured from the standard healthy porcine model for brain (n = 5) and pancreatic (n = 4) tissues measured at 0.167 ± 0.016 S/m and 0.188 ± 0.02 S/m, respectively (Fig. 8A). A t-test was used to determine statistically significant differences between the *RAG2/IL2RG* pig healthy tissue and the standard porcine model healthy tissue; no statistical differences were detected for brain tissue (*p* = 0.4401), but a statistical difference was detected for the pancreatic tissue (*p* = 0.021). High frequency conductivity values for healthy tissue analyzed from the *RAG2/IL2RG* pig model for brain (n = 8), pancreas (n = 3), and prostate (n = 7) tissue measured 0.294 ± 0.021 S/m, 0.371 ± 0.009 S/m, and 0.508 ± 0.043 S/m (Fig. 8A). In this same frequency, conductivity of the standard healthy porcine model for brain (n = 5) and pancreatic (n = 4) tissue measured at 0.276 ± 0.016 S/m and 0.383 ± 0.009 S/m, respectively. Due to the lack of easily accessible, normal porcine prostatic tissue, we compared SCID-like prostate conductivity to that collected from freshly excised human prostates and reported in Halter et. al, 2007^42^. Halter and colleagues measured low- and high-frequency conductivities of 0.43 ± 0.27 S/m and 0.60 ± 0.32 S/m, respectively, neither of which are significantly different (*p* = 0.514 and *p* = 0.551) than the values reported herein for SCID-like prostate conductivity (Fig. 8A). A t-test was used to determine statistically significant differences between the *RAG2/IL2RG* healthy tissue and the standard porcine model healthy tissue; no statistical differences were detected for brain (*p* = 0.1092) or pancreatic tissue (*p* = 0.1349) across porcine models. Due to the lack of healthy porcine prostate data available, no direct comparisons were made between the SCID-like prostate and conventional porcine prostate tissue at the abovementioned frequencies.

## Discussion

With their larger size compared to rodents and controlled immune system knockdown, *RAG2/IL2RG* pigs created with the CRISPR/Cas9 system may be used as a novel oncology model for translating developing therapies. They offer the opportunity to grow xenograft tumors from immortalized cell lines, as well as, primary tumors from patients, in an organism that can grow to a scale comparable to the size of humans. Previous animal models have been limited due to small animal size, preventing the development of tumors that are comparable in size and location to humans, and other large animal models are less predictable and reproducible. With this controlled immunocompromised model, the capability to engraft tumors would allow for more relevant and efficient pre-clinical trials.

The growth of the tumors in the subcutaneous space in the pigs was notable immediately after engraftment and maintained a relatively steady growth rate beyond that point. This controlled growth in conjunction with histology, which showed no signs of host immune rejection from the myeloid cells that remain in immunocompromised conditions, indicate that this model is capable of supporting xenografts even with the moderate cell injections of 1.2 × 10^6^ used here. The current study stopped tumor growth at 36 days as a pre-determined end point for consistency and direct comparisons with the mouse models, not due to health effects or tumor burden, which commonly prevent murine models from producing larger tumors. Beyond that, should this model be used for subcutaneous treatments, the immunocompromised state of the pigs did not leave them susceptible to increased systemic disease, as seen by their steady increase in weight, lack of clinical symptoms, and their lack of metastases found on necropsy. Future studies should be conducted in order to determine if orthotopic engraftment can be supported by the animals without notable health effects.

General comparison of the porcine xenografted Panc01 tumors to the murine Pan02 tumors and PDAC murine xenografted PDX tumors, is based upon the latter two models’ well-established roll in studying cancer therapies^24,38–40,43^. Based on histology, the xenograft Panc01 tumors share comparable features of interest with the accepted PDX human-tumor xenografted models. The PDX tumors are grown in NSG, immunocompromised, mice, and develop glandular structures that express CK19, a molecule found in nearly all cases of pancreatic adenocarcinoma^41,44^. These glands often have distinct lumens and will often be filled with clear space, small amounts of secretory product, inflammatory cells, and/or cellular debris. The most common type of pancreatic tumor in humans is pancreatic ductal adenocarcinoma, which is histologically characterized by polygonal cells often arranged into glandular structures^45^. Similar to the PDX models, the Panc01 tumors from the immunocompromised pigs also closely resemble this microscopic morphology and are consistent with observations in human patients. This includes cells with marked pleomorphism and prominent mitotic figures. Another feature of the tumors in PDX mice is the fairly prominent fibrovascular stroma, which is not present in the Pan02 tumors. However, the pig tumors do exhibit an intermediate stroma again suggesting it is more similar to the PDX than the Pan02. The tumor stroma seems to be a pretty important player and is often a barrier to treatment, making the Panc01 tumors physiologically relevant for preclinical testing^46–48^. Additionally, agreeing with our reported pathologic evaluation that there are no ductal formations and with previous studies, we did not find ductal cell maker CK19 on our Pan02 tumors^49^. On the other hand, the murine PDX and porcine Panc01 tumors both express this marker, demonstrating both models similarity to PDAC tumors^41,44,50^. These comparisons, between Panc01 and Panc02 tumors, indicate that the pig tumors formed by Panc01 cells are more appropriate than the Panc02 model previously used for IRE analyses. However, as different cells lines may grow tumors with different characteristics, a broader study employing additional cell lines should be conducted to confirm this relationship between other human, porcine, and murine tumors. Thus, this model recapitulates many features found in patient adenocarcinoma and is comparable to PDX tumors. While there are other cell lines of pancreatic adenocarcinoma and variety in the phenotype found in clinical patients, the data presented here shows that the Panc01 tumors grown in immunocompromised pigs is comparable in histological phenotype to two other, well established pancreatic cancer models and is thus a sufficient model for pre-clinical studies.

For the feasibility studies described here, we used IRE as an example technology that can benefit from tumors propagated in the *RAG2/IL2RG* pigs. Due to electroporation-based therapies relying on exposing targeted cells to a specific electric field, the conductivities of the tissues in the region of interest are critical to determining treatment outcome. Thus, conductivity is one of the principal parameters affecting the production of lesions due to irreversible electroporation, and preclinical animal models must exhibit similar electrical signatures to their human counterparts to maximize clinical translatability. Additionally, the morphology of cells affects the local electric potential they experience when an external electric field is applied^51–53^. Thus, faithful recapitulations of human tumor tissue should be similar in terms of cell shape, size, distribution, and intracellular content as this will ultimately affect the treatment outcome. Notably, the PDX tumors presented in this study have been shown to recapitulate the histological characteristics of human PDAC samples^24^. Thus, the similar morphology of cells in the Panc01 tumors introduced here indicates similarity to primary tumor samples as well. Though the Panc01 tumors exhibited fewer ductal structures than the human PDX tissue, this did not introduce differences in measured electrical properties. This similarity is likely the result of the similar cell size and packing density between the PDX and Panc01 tissue (Fig. 5). However, it is possible that the relatively large samples (~ 0.2 cm^3^), excision process, and PBS wash introduce minute changes in tissue structure/composition that limit the ability of our methods to resolve small spatial differences in conductivity due to the presence of these ductal structures. Despite this, we have shown that the bulk tissue response is similar between tissue models, suggesting that the Panc01 platform exhibits a similar bulk response to applied fields as currently established tissue surrogates.

To assess the electrical similarity of the subcutaneous Panc01 tumors to locally advanced pancreatic cancer (LAPC) observed in humans, we conducted a series of experiments in which electrical pulses were delivered across flat-plate electrodes to determine electrical conductivity as a function of applied electric field. Our results demonstrate that the Panc01 tumors grown in *RAG2/IL2RG* pigs have similar electrical behavior to spontaneous tumors in humans, as well as murine-propagated human PDAC tissue, increasing our confidence that the local electric field produced in the pig-derived tumors will be similar to that which arises in human neoplasms.

These data support the feasibility of the immunocompromised pig model for evaluating the clinical relevance of novel medical devices and other surgical procedures, including those that rely on accurate recapitulation of human tumor morphology and electrical properties. Here we have shown for the first time that the dielectric properties of *RAG2/IL2RG* pigs are similar to those of humans. Future studies are needed to establish the growth of tumors implanted orthotopically for more ideal pre-clinical treatment protocols utilizing this physiologically relevant oncology model. Overall, these data indicate that this immunocompromised model is capable of recapitulating physiologically relevant pancreatic tumors comparable to other commonly utilized tumor models with several novel advantages and with the potential to be utilized to study other malignancies. Together, this suggests that this model will be highly useful and capable of bridging the gap of translating ablation therapies from the bench to clinical application.

## Materials and methods

### Generation of immunodeficient pigs

Oocytes aspirated from sow ovaries were matured in maturation medium (TCM-199 medium supplemented with 0.5 IU ml^−1^ FSH, 0.5 IU ml^−1^ LH, 0.82 mM cysteine, 3.02 mM glucose, 0.91 mM sodium pyruvate and 10 ng ml^−1^ EGF) for 40–44 h. After in vitro maturation, cumulus cells were removed by vortexing in the presence of hyaluronidase, then mature oocytes extruding the first polar body were transferred to fertilization media (modified Tris-buffered medium supplemented with 113.1 mM NaCl, 3 mM KCl, 7.5 mM CaCl_2_, 11 mM glucose, 20 mM Tris, 2 mM caffeine, 5 mM sodium pyruvate and 2 mg ml^−1^ BSA). Extended semen was washed with PBS, then introduced into the IVF dishes containing oocytes. The gametes were co-incubated for 5 h at 38.5 °C and 5% CO_2_. Microinjection was conducted in manipulation media (TCM199 with 0.6 mM NaHCO_3_, 2.9 mM HEPES, 30 mM NaCl, 10 ng ml^−1^ gentamicin and 3 mg ml^−1^ BSA) covered with mineral oil on the heated stage of a Nikon inverted microscope (Nikon Corporation, Tokyo, Japan). Two sgRNAs targeting the *RAG2* gene, three sgRNAs targeting the *IL2RG* gene, and Cas9 mRNA (10 ng/µl sgRNA each and 20 ng/μl Cas9 mRNA) were introduced into the cytoplasm of the presumable zygotes using a FemtoJet microinjector (Eppendorf, Hamburg, Germany). Target sequences of the sgRNAs can be found in Table 1. The injected embryos were moved to culture media supplemented with 10 ng ml^−1^ GM-CSF and incubated at 38.5 °C, 5% CO_2_, and 5% O_2_ until embryo transfer^54^. The cultured embryos were transferred into a surrogate gilt at day 4 post-IVF by surgically transferring into the oviduct. Pregnancy was determined by ultrasound at around day 30 of gestation. To genotype the mutations generated by CRISPR/Cas9 system, genomic DNA was isolated from tail-tips of the newborn piglets using PureLink Genomic DNA kit (Thermo Fisher Scientific) following the manufacturer’s instructions. Primers flanking projected double strand break (DSB) sites were designed to amplify target region of *RAG2* and *IL2RG* genes (Table 2). The target regions were PCR amplified using Dream Taq DNA Polymerase (Thermo Fisher Scientific). PCR conditions were as follows: initial denature at 95 °C for 2 min; denature at 95 °C for 30 s, annealing at 60 °C for 30 s, and extension at 72 °C for 30 s for 34 cycles; 72 °C for 5 min; and holding at 4 °C. The PCR amplicons were sequenced to determine the mutation types generated by the CRISPR/Cas9 system. Immunocompromised state was verified with flow cytometry to analyze the presence B (CD79A), T (CD3E), and NK (CD3E-/CD56 +) cells.

**Table 1:** Target sequences of *RAG2* and *IL2RG* sgRNAs.

Target gene	Sequence (PAM)
*RAG2*	5′-GAATGACCATATCTGCCTTC(AGG)
	5′-GTATAGTCGAGGGAAAAGTA(TGG)
*IL2RG*	5′-CCAACCTAACTCTGCACTAC(TGG)
	5′-TGGAAACGTTGAGAGTCCC(AGG)
	5′-AAACGTTGAGAGTCCCAGG(GGG)

**Table 2:** Primers used to amplify sgRNA target regions.

Primer	Sequence
RAG2_Forward	GGCTTTCCCATAACCTGGATATTGGGTC
RAG2 Reverse	CGTCTCAGACTCATCTTCCTCATCATCTT
IL2RG_Forward	CGGTAATAATCATGACTAGAGGGAATGAAAGATTGATTTATC
IL2RG_Reverse	GAGAGAAAGTTGGGTGTCTATAAAAGAAGGGAGAATTAAAACTG

### Animal care

Six pigs (n_total_ = 5 males and n_total_ = 1 female; n = 2 male and n = 1 female used for the current study) were delivered through a sterile hysterectomy process previously described by a board-certified large animal veterinarian^55^. Pigs were monitored regularly until fully recovered from anesthesia and able to eat independently. Tail snips were taken from each piglet while still under the influence of anesthesia for genotyping, and ears were clipped for numbering. The pigs were born into sterile, gnotobiotic isolators and were fed sterile boxed milk throughout the study in order to prevent infections^55^. All animal experiments were approved and carried out in accordance with the Virginia Tech Institutional Animal Care and Use Committee under IACUC protocol: 19-117-CVM.

### Tumor injection and monitoring

Human Panc01 cells (National Cancer Institute DTP, DCTP Tumor Repository) were grown up in DMEM supplemented with 10% FBS and 1% penicillin/ streptomycin and removed from plates with Trypsin in EDTA. While the pigs were still under the effect of anesthesia from the hysterectomy, 1.2 × 10^6^ Panc01 cells in 100 µL of Matrigel (Corning, USA) were injected subcutaneously behind the ears of three pigs, with an additional 100 µL of Matrigel behind the left ear. Tumors were measured three times per week with plastic Vernier calipers. Diameter was calculated as the square root of the product of the widest diameter and the diameter perpendicular to that. At the same point of tumor measurement, weight and other health monitoring was also completed. Following euthanasia, animals were necropsied and tissues were fixed in 10% formalin for at least 24 h. Paraffin-embedded formalin-fixed tissues were H&E stained and evaluated by board-certified veterinary pathologist.

### Immunohistochemistry

Pan02, PDX, and Panc01 samples (n = 3/group) were subject to IHC-P staining of CK19 (Thermo Fisher, MA5-12,663). Paraffin-embedded formalin-fixed tissue sections were deparaffinized following established xylene:ethonal wash protocol^56^. Antigen retrieval was achieved through 1-h incubation at 95 °C on a hot plate in sodium citrate buffer with pH6 based upon the antibody manufacture’s suggestion. Once cooled, slides were washed twice in tris-buffered saline (TBS) plus 0.025% Triton X-100 with gentle agitation. Primary antibody was diluted (1:250) in TBS with 1% BSA and 10% FBS overnight at 4 °C. Slides were washed four times in tris-buffered saline (TBS) plus 0.025% Triton X-100 with gentle agitation, prior to 2 h room temperature incubation with secondary antibody (Cell Signaling, 7076P2) diluted (1:2000) in TBS with 1% BSA and 10% FBS. Slides were washed three times for 5 min in TBS, then developed with DAB substrate (Abcam, ab64238) following manufactures guidelines and counterstained with eosin and methyl blue.

### Ultrasound imaging

Animals were anesthetized with Xylazine/Telazol. Prior to imaging, pigs were cleaned and dried, and had the area of interest shaved to remove hair that could interfere with imaging. Ultrasound imaging was done using both linear and phased array ultrasound transducers (L18-10L30H-4 and P8-3L0S1-6, TELEMED, Lithuania, EU). Images were acquired on a laptop computer and saved for future analysis. Targets were confirmed by a board-certified veterinarian.

### Tissue handling and electroporation

Small rectangular sections of healthy or malignant tissue were excised, washed to prevent blood coagulation, and placed in modified PBS to help maintain osmotic pressure balance as previously described^57^. Immediately prior to pulsing, a sample of the tissue was removed from PBS, sectioned into smaller pieces, and placed into an insulating cylindrical polydimethylsiloxane (PDMS) mold as previously described^58^. The mold had radius of 3 mm and thickness of 0.56 cm. The tissue-containing mold was placed between a set of parallel plate electrodes connected to a pulse generator (BTX ECM 830, Harvard Apparatus, Holliston, MA). Baseline electrical conductivity was measured by delivering a low voltage (~ 20 V) potential across the electrodes. Electrical impedance was then recorded from 1 to 10^6^ Hz.

All excised tumor and healthy tissues were analyzed within 1 h of resection. Prior to data analysis, all DC offsets were removed and a Butterworth filter was applied to smooth the data and allow for automated analysis in MATLAB (MathWorks Inc., Natick, MA, USA). To assess differences in electrical properties between our *RAG2/IL2RG* pig Panc01 tumor model and primary human pancreatic cancer specimen, we compared the initial tissue conductivities and the changes in tissue conductivity at 2.5 kV/cm. Baseline electrical properties were recorded for healthy pancreas, prostate and brain tissue excised from *RAG2/IL2RG* pigs. Prior to IRE delivery, a low voltage (~ 20 V) pulse was administered to record sample-specific initial conductivity. This low potential pulse does not cause electroporation-induced changes in electrical properties, and is thus only diagnostic. The resulting value was used as a baseline to determine percent differences in tissue conductivity during the subsequent high voltage, 100-pulse IRE protocol. An electric field strength of either 500, 750, 1000, 1500, 2000, 2500, and 3000 V/cm was delivered to each sample (n = 1 for each electric field strength, n = 7 total samples). For comparison, the data acquired in this study are reported alongside tissue conductivity data from primary human pancreatic cancer, as previously reported in Beitel-White et al., primary pancreatic ductal adenocarcinoma (PDAC) tissue propagated in mice from Brock et al*.*, as well as, data obtained from literature for healthy pancreas, prostate, and brain tissue^24,42,59–65^.

To monitor changes in temperature due to joule heating, a general-purpose STB fiber optic thermometry probe (LumaSense, Santa Clara, CA, USA) was inserted at the midpoint of the cylindrical tissue sample. Temperature was recorded at a frequency of 2 Hz using a Luxtron m3300 Biomedical Lab Kit (LumaSense, Santa Clara, CA, USA). An unpaired t-test with Welch’s correction was used to compare the mean initial conductivity.

## Supplementary Information

Supplementary Information

## Abbreviations

SCID: Severe combined immunodeficiency
IRE: Irreversible electroporation
PDX: Patient derived xenograft
