# Supplementary material for: Establishing an immunocompromised porcine model of human cancer for novel therapy development with pancreatic adenocarcinoma and irreversible electroporation
Source: Sci Rep. 2021 Apr 7;11:7584. doi: 10.1038/s41598-021-87228-5 (PMC8027815; doi:10.1038/s41598-021-87228-5)

Establishing an Immunocompromised Porcine Model of Human Cancer for Novel Therapy Development with Pancreatic Adenocarcinoma and Irreversible Electroporation

Alissa Hendricks-Wenger[2,3,8], Kenneth N. Aycock[2], Margaret A. Nagai-Singer[3], Sheryl Coutermarsh-Ott[3], Melvin F. Lorenzo[2], Jessica Gannon[2,6], Kyungjun Uh[1], Kayla Farrell[1], Natalie Beitel-White[4], Rebecca M. Brock[3,8], Alexander Simon[2], Holly A. Morrison[3], Joanne Tuohy[7], Sherrie Clark-Deener[5], Eli Vlaisavljevich[2,8,9], Rafael V. Davalos[2,6,8,9] , Kiho Lee[1], Irving C. Allen[3,8,9,10*]

[1] Department of Animal and Poultry Sciences, College of Agriculture and Life Sciences, Virginia Tech, Blacksburg, VA 24061, USA
[2] Department of Biomedical Engineering and Mechanics, Virginia Polytechnic Institute and State University, Blacksburg 24061, VA, USA
[3] Department of Biomedical Sciences and Pathobiology, Virginia-Maryland College of Veterinary Medicine, Blacksburg 24061, VA, USA
[4] Department of Electrical and Computer Engineering, Virginia Polytechnic Institute and State University, Blacksburg, VA 24061, USA
[5] Department of Large Animal Clinical Sciences, Virginia-Maryland College of Veterinary Medicine, Blacksburg, VA 24061, USA
[6] Department of Mechanical Engineering, Virginia Polytechnic Institute and State University, Blacksburg, VA 24061, USA
[7] Department of Small Animal Clinical Sciences, Virginia-Maryland College of Veterinary Medicine, Blacksburg, VA 24061, USA
[8] Graduate Program in Translational Biology, Medicine and Health, Virginia Polytechnic Institute and State University, Roanoke 24016, VA, USA
[9] Institute for Critical Technology and Applied Sciences Center for Engineered Health, Virginia Tech, Kelly Hall, Blacksburg, VA 24061, USA
[10]Department of Basic Science Education, Virginia Tech Carilion School of Medicine, Roanoke, 24016, VA, USA

Supplemental Figure S1. Genotyping of piglets carrying modified IL2RG and RAG2 gene.

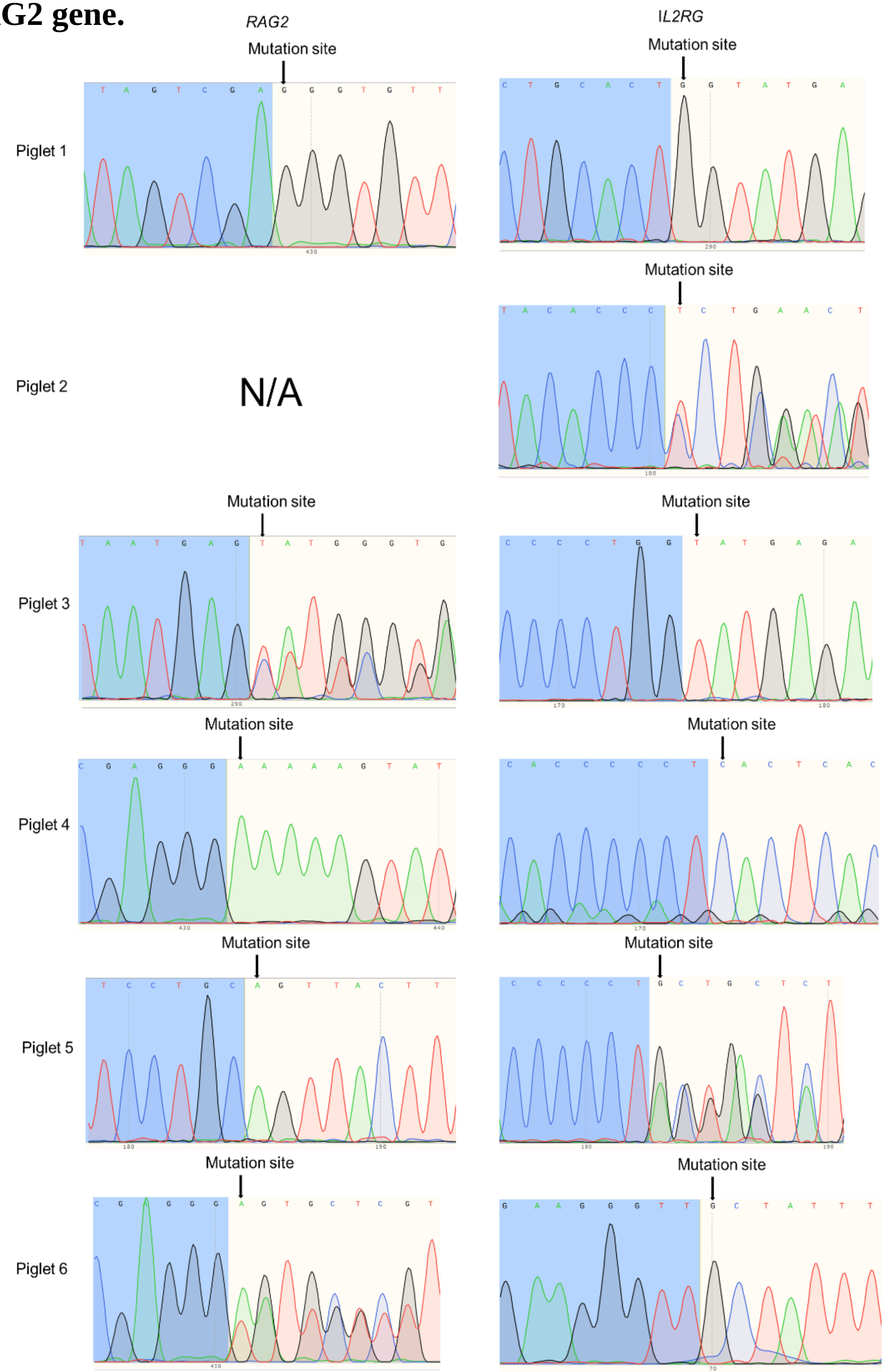

Supplement: Supplementary file 1 — Supplementary Information [file 41598_2021_87228_MOESM1_ESM.pdf]
